# Phosphocalcic Markers and Calcification Propensity for Assessment of Interstitial Fibrosis and Vascular Lesions in Kidney Allograft Recipients

**DOI:** 10.1371/journal.pone.0167929

**Published:** 2016-12-30

**Authors:** Lena Berchtold, Belen Ponte, Solange Moll, Karine Hadaya, Olivia Seyde, Matthias Bachtler, Jean-Paul Vallée, Pierre-Yves Martin, Andreas Pasch, Sophie de Seigneux

**Affiliations:** 1 Service of Internal Medicine, Department of Internal Medicine, University Hospital of Geneva, Geneva, Switzerland; 2 Service of Nephrology, Department of Internal Medicine Specialities, University Hospital of Geneva, Geneva, Switzerland; 3 Institute of Clinical Pathology, Departement of Clinical Pathology, University Hospital of Geneva, Geneva, Switzerland; 4 Service of Clinical Research, Department of Clinical Research, University of Bern, Bern, Switzerland; 5 Service of Radiology, Department of Radiology and Medical Informatics, University Hospital of Geneva, Geneva, Switzerland; "INSERM", FRANCE

## Abstract

Renal interstitial fibrosis and arterial lesions predict loss of function in chronic kidney disease. Noninvasive estimation of interstitial fibrosis and vascular lesions is currently not available. The aim of the study was to determine whether phosphocalcic markers are associated with, and can predict, renal chronic histological changes. We included 129 kidney allograft recipients with an available transplant biopsy in a retrospective study. We analyzed the associations and predictive values of phosphocalcic markers and serum calcification propensity (T_50_) for chronic histological changes (interstitial fibrosis and vascular lesions). PTH, T_50_ and vitamin D levels were independently associated to interstitial fibrosis. PTH elevation was associated with increasing interstitial fibrosis severity (r = 0.29, p = 0.001), while T_50_ and vitamin D were protective (r = -0.20, p = 0.025 and r = -0.23, p = 0.009 respectively). On the contrary, fibroblast growth factor 23 (FGF23) and Klotho correlated only modestly with interstitial fibrosis (p = 0.045) whereas calcium and phosphate did not. PTH, vitamin D and T_50_ were predictors of extensive fibrosis (AUC: 0.73, 0.72 and 0.68 respectively), but did not add to renal function prediction. PTH, FGF23 and T_50_ were modestly predictive of low fibrosis (AUC: 0.63, 0.63 and 0.61) but did not add to renal function prediction. T_50_ decreased with increasing arterial lesions (r = -0.21, p = 0.038). The discriminative performance of T_50_ in predicting significant vascular lesions was modest (AUC 0.61). In summary, we demonstrated that PTH, vitamin D and T_50_ are associated to interstitial fibrosis and vascular lesions in kidney allograft recipients independently of renal function. Despite these associations, mineral metabolism indices do not show superiority or additive value to fibrosis prediction by eGFR and proteinuria in kidney allograft recipients, except for vascular lesions where T50 could be of relevance.

## Introduction

Renal interstitial fibrosis (IF) and arterial lesions are predictive of loss of renal function in chronic kidney disease (CKD) [1–3]. Kidney allografts are very prone to develop IF and vascular lesions secondary to acute or chronic rejection, and to calcineurin inhibitors toxicity [4–6]. Currently, IF and arterial lesions are evaluated by histopathology which necessitates kidney biopsies [7–10]. There are however many limitations to histopathological assessment, such as sampling error or bias, semi-quantitative and poorly reproducible scoring, biopsy complications and cost of the procedure [11]. Established noninvasive tools to estimate kidney IF and vascular lesions are currently not available. These would be very helpful for the evaluation of the whole organ without the inherent risks associated to repeated biopsies. Indeed, a better non-invasive appreciation of the formation of IF and arterial lesions in patients would allow earlier treatment adaptation and better follow-up.

Amongst potential tools, phosphocalcic biomarkers are of interest. Disorders in phosphorus and calcium metabolism are common in CKD. In addition to the traditional markers such as calcium, phosphate, parathyroid hormone (PTH) and vitamin D levels, new biomarkers such as Klotho and fibroblast growth factor 23 (FGF23) are emerging. Klotho is a protein mainly expressed in kidney tubular cells. During early phases of experimental CKD, Klotho expression is downregulated [12, 13]. In humans, this early decrease in soluble Klotho can be measured [14]. Klotho downregulation plays an important role in kidney fibrosis progression by different modalities including repression of the WNT pathway [15–18]. In addition, lower Klotho levels are associated with higher prevalence of cardiovascular disease, arterial stiffness and vascular calcification in the experimental setting and in some clinical observations [15, 17, 19, 20]. This could be related to Klotho’s direct phosphaturic properties [13] and its role as an FGF23 receptor cofactor. Klotho loss may therefore be a sensitive marker for nephron loss, early fibrosis formation and could also be an interesting marker of chronic vascular lesions.

FGF23 is a key phosphaturic hormone produced by osteocytes and osteoblasts that increase early in CKD [21, 22]. The cause of this elevation is still debated, but may result from phosphate retention, Klotho loss, and kidney production of FGF23 or abnormal bone regulation. FGF23 is therefore a sensitive marker of kidney disease and cardiovascular complications in CKD. Whether FGF23 indicates chronic kidney histological changes has not been studied so far. All parameters of mineral metabolism are to some extent related: suppression of Klotho increases FGF23 levels, which in turn suppresses vitamin D. Increased phosphate, FGF23 and parathyroid hormone (PTH) are independent risk factor for CKD progression and cardiovascular mortality not only in primary CKD but also in kidney allograft recipients [21, 23–40]. However, whether mineral metabolism components correlate with IF and vascular lesions and could be earlier markers than eGFR for histological lesions is not known.

In addition to these new biomarkers, a blood test was recently described that measured the blood calcification propensity by monitoring the maturation time (T_50_) of calciprotein particles in serum [41]. High calcification propensity (or low T_50_) was closely associated with progressive aortic stiffening and increased long-term mortality in CKD patients [42, 43]. Surprisingly, T_50_ was also predictive of renal function loss in kidney allograft recipients [44]. However, this test has not yet been studied in association with renal histological lesions.

Due to the lack of data on the relation between phosphocalcic markers and chronic histological changes, we aimed to assess this association in a retrospective study. We analyzed the associations and predictive values of phosphocalcic markers and T_50_ with chronic histological changes (IF and vascular lesions) in kidney allograft recipients undergoing protocol or clinically-driven biopsies. We hypothesized that phosphate, FGF23, PTH, T_50_, Klotho and vitamin D (25D) levels may be useful markers of early IF and chronic vascular lesions in this population, independently of eGFR.

## Results

### Characteristics of the study population

We included 129 kidney allograft recipients, mainly Caucasian (95%) and male (60%). Baseline characteristics are presented in Table 1. The main primary kidney diseases were glomerulonephritis and hypertensive nephropathy. Median graft vintage was 5 years. eGFR was below 60ml/min/1.73m2 in 65% of patients and office blood pressure was ≥140/90mmHg in 44%. Overweight patients represented 33% of the population whereas 17% were obese (BMI≥30kg/m^2^). Mean sodium, potassium, bicarbonate, albumin, calcium and phosphate levels were within population-based reference intervals and considered well controlled. Serum PTH was significantly higher than the normal range. Almost all patients were on 25-hydroxyvitamin D and calcium supplements (88% and 71% respectively). The immunosuppressive regimen was mainly composed of calcineurin inhibitors (88.4%: 68.2% of tacrolimus and 20.2% of ciclosporine), mycophenolate mofetil (78.3%) and steroids (60.5%). Only 12.4% of patients were on azathioprine and 1.6% on m-Tor inhibitors.

**Table 1:** Baseline characteristics of the study population (n = 129): clinical parameters, medication and laboratory measurements.

Characteristics	Value
**Clinical parameters**	
Age, years	57.0 (46.0–69.1)
Male, n (%)	78 (60)
Body mass index, kg/m^2^	25.3 ± 4.6
Caucasian, n (%)	123 (95)
Deceased donor transplant, n (%)	80 (62)
History of acute rejection, n (%)	49 (38)
Graft vintage, years	5.0 (2.0–12.0)
Systolic BP, mmHg	132 ± 17
Diastolic BP, mmHg	79 ± 12
Current smoker, n (%)	12 (9.3)
Etiology of kidney disease, n (%)*	
Diabetes	8 (6.2)
Hypertension	27 (20.9)
Glomerulonephritis	29 (22.5)
Polycystic kidney disease	24 (18.6)
Others (tubulointerstitial nephritis, reflux, …)	68 (52.7)
**Medication, n (%)**	
ACEi/ARB	66 (51.2)
Calcium channel blockers	57 (44.2)
Diuretics	14 (10.9)
Beta-blockers	72 (55.8)
Statins	85 (66.9)
Calcium supplementation	91 (70.5)
1.25OH-vitamin D supplementation	15 (11.6)
25OH-vitamin D supplementation	113 (87.6)
**Laboratory measurements**	
Creatinine, micromol/l	128 ± 47
eGFR ml/min per 1.73m^2^ ^**^	55 ± 20
Albumin, g/l	37.2 ± 3.5
Corrected calcium, mmol/l	2.4 ± 0.14
Phosphate, mmol/l	1.1 ± 0.2
Magnesium, mmol/l	0.72 ± 0.1
25-hydroxyvitamin D, nmol/l (n = 125)	68 ± 22
Parathyroid hormone, pmol/l(n = 126)	8.5 (6.0–10.8)
Hemoglobin, g/l	129 ± 15
FGF23, RU/ml	39.3 (27.7–54.6)
Klotho, pg/ml	744 ± 246
T_50_, min	285 ± 61
Spot urine protein/creatinine ratios, g/24h (n = 119)	0.12 (0.07–0.25)
Albuminuria, n (%)	
• <30 mg/g	55 (42.6)
• 30-300mg/g	51 (39.5)
• >300mg/g	23 (17.8)

Values reported as numbers and %, mean±SD, or median (interquartile ranges) as appropriate.

*One patient may have more than one etiology to their kidney disease.

**eGFR (estimated Glomerular Filtration Rate) was calculated according to the Chronic Kidney Disease Epidemiology Collaboration equation.

ACEi/ARB, angiotensin-converting enzyme inhibitor/angiotensin II receptor blocker.BP: Blood Pressure; FGF23: Fibroblast growth factor 23; T_50_: Calcification propensity.

Indications and results from kidney biopsies are presented in Table 2. Twenty-three percent of patients had ≤10% fibrosis, 55.0% had ≤20%, 76.0% had ≤30% and 9.3% had >40%. Significant vascular lesions (BANFF: cv+ah+aah>5) were present in 36.4% of patients. Von Kossa staining was available from 88 patients, among which 8 (9%) were positive for mineral deposits.

**Table 2:** Baseline characteristics of the study population (n = 129): biopsy indication, biopsy diagnosis and chronic histological lesions.

Characteristic	Value
**Biopsy indication**	
Protocol biopsies	115 (89.1)
Creatinine increase	6 (4.7)
De Novo Proteinuria	4 (3.1)
Increase in creatinine and proteinuria	3 (2.3)
De Novo Donnor Specific Antibodies	1 (0.8)
**Biopsy diagnosis,** n (%)*	
Rejection	13 (10.1)
Tubulo-interstitial lesions	10 (7.8)
Immunological glomerulonephritis	22 (17.1)
Diabetic nephropathy	7 (5.4)
Hypertensive nephropathy	6 (4.7)
Anticalcineurin toxicity	36 (27.9)
Chronic allograft nephropathy	4 (3.1)
Others (vascular lesions, …)	31 (24)
**Chronic Histological lesions**	
Fibrosis in %	24.3 ± 15.7
BANFF score	
• IF/TA^a^ (ci + ct), min 0 –max 6	2 (2–4)
• Vascular lesions (cv + ah + aah), min 0 –max 9	3 (1–4)
Von Kossa (%)	
• Positive, n (%)	8 (9)

Values reported as number and %, mean±SD, or median (interquartile ranges) as appropriate.

* One biopsy may have more than one diagnosis.

^a^Interstitial fibrosis and tubular atrophy.

ah: arteriolar hyaline thickening; aah: circumferential hyaline arteriolar thickening; cv: vascular fibrous intimal thickening.

All mineral metabolism markers correlated well with eGFR except calcium, PTH and 25D (S1 Fig).

### Association of phosphocalcic biomarkers and T_50_ with interstitial fibrosis

**Fig 1:**
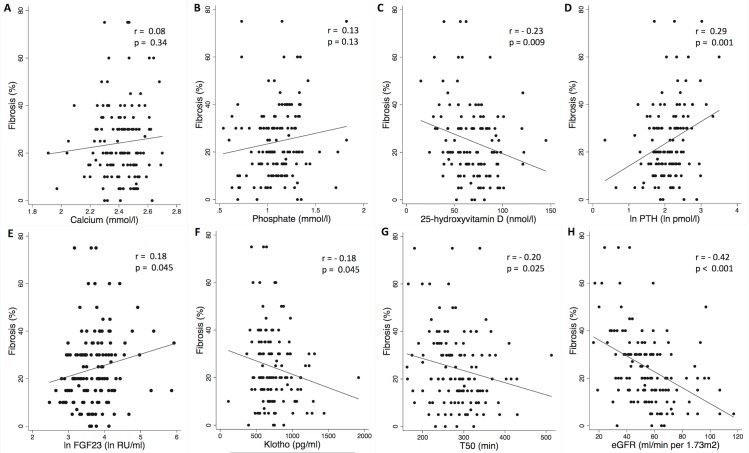
Correlations between phosphocalcic biomarkers, T_50_ and interstitial fibrosis in renal allograft recipients (n = 129). Scatter plot graphs of (A) calcium, (B) phosphate, (C) vitamin D, (D) ln PTH, (E) ln FGF23, (F) Klotho, (G) T_50_, (H) eGFR versus interstitial fibrosis. Each symbol represents one patient. The continuous line indicates least-square linear regression. eGFR: estimated Glomerular Filtration Rate; FGF23: Fibroblast growth factor 23; PTH: parathyroid hormone; T_50_: Calcification propensity; Ln: log-transformed.

We studied the associations of calcium, phosphate, 25D, PTH, FGF23, Klotho and T_50_ with histological fibrosis. Fig 1 shows the linear trends and correlation coefficients. The percentage of fibrosis increased with rising FGF23 and PTH levels. On the contrary, IF increased with decreasing levels of Klotho, T_50_ and 25D. eGFR, creatinine and proteinuria were well correlated to IF (Fig 1 and S2A and S2B Fig). Serum calcium and phosphate were not correlated with IF. Results were not significantly modified by the exclusion of non protocol biopsies (S4 Fig). When fibrosis was considered in three categories determined by Banff scoring for chronic allograft nephropathy (0–25; 26–50; >50% of intertistitial fibrosis), mineral metabolism markers were well associated to fibrosis progression (S3 Fig).

Table 3 displays models of linear regression analyses. Calcium was not associated with IF neither in univariate nor in multivariate analyses. Phosphate was not associated with IF in the univariate analysis but was associated in multivariate analysis. The borderline association of Klotho and FGF23 with IF disappeared after adjustment for the other mineral metabolism parameters. Conversely, the association between phosphate and IF disappeared after adjustment for eGFR only. T_50_ remained associated with IF after adjustement for other mineral metabolism parameters and eGFR but this association was attenuated after adjustment for proteinuria. Only 25D and PTH remained independently associated with IF after all multivariate analysis. Since in transplanted patients, tertiary hyperparathyroidism is relatively common, in order to rule out the possibility that hyperparathyroidism might influence the correlation between PTH or 25D with fibrosis, we excluded patients with tertiary hyperparathyroidism (n = 13). In this case our results were unchanged.

**Table 3:** Associations of serum biomarkers with interstitial fibrosis in kidney allograft recipients (n = 129). Univariate and multivariate linear regression analysis.

Biomarkers	Coefficient	95% CI	p Value
**Calcium (mmol/l)**			
Unadjusted	9.40	-10.0 to 28.8	0.34
Model 1^a^	9.61	-9.72 to 28.9	0.33
Model 2	9.03	-10.5 to 28.6	0.36
Model 3	9.55	-9.15 to 28.3	0.31
Model 4	10.1	-10.1 to 30.2	0.32
**Phosphate (mmol/l)**			
Unadjusted	8.96	-2.71 to 20.6	0.131
Model 1^b^	19.0	6.75 to 31.3	0.003
Model 2	16.0	3.26 to 28.7	0.014
Model 3	11.1	-1.28 to 23.5	0.078
Model 4	12.4	-1.11 to 25.9	0.072
**Ln PTH (pmol/l),** n = 126			
Unadjusted	9.36	3.81 to 14.9	0.001
Model 1^c^	10.8	4.79 to 16.7	0.001
Model 2	9.14	2.87 to 15.4	0.005
Model 3	7.68	1.58 to 13.8	0.014
Model 4	7.59	0.98 to 14.2	0.025
**VitaminD (nmol/l),** n = 125			
Unadjusted	-0.16	-0.29 to -0.04	0.009
Model 1^d^	-0.12	-0.24 to 0.002	0.054
Model 2	-0.14	-0.26 to -0.02	0.024
Model 3	-0.15	-0.27 to -0.03	0.012
Model 4	-0.15	-0.27 to -0.02	0.022
**Klotho (pg/ml)**			
Unadjusted	-0.01	-0.02 to 0.0002	0.045
Model 1	-0.01	-0.02 to 0.002	0.129
Model 2^e^	-0.01	-0.02 to 0.003	0.161
Model 3	-0.005	-0.02 to 0.006	0.374
Model 4	-0.005	-0.02 to 0.007	0.426
**Ln FGF23 (RU/ml)**			
Unadjusted	4.69	0.10 to 9.28	0.045
Model 1	2.73	-1.99 to 7.45	0.255
Model 2^f^	2.36	-2.38 to 7.09	0.327
Model 3	-0.51	-5.31 to 4.29	0.834
Model 4	-0.51	-5.54 to 4.52	0.841
**T**_**50**_ **(min)**			
Unadjusted	-0.05	-0.10 to -0.01	0.025
Model 1^g^	-0.06	-0.11 to -0.02	0.005
Model 3^h^	-0.04	-0.09 to -0.003	0.036
Model 4	-0.04	-0.09 to 0.0005	0.053

Model 1: adjusted for calcium, phosphate, log PTH, vitamin D; Model 2: adjusted for model 1 and Klotho and FGF23; Model 3: adjusted for model 2 and diabetes, HTA, graft vintage and eGRF; Model 4: adjusted for model 3 and proteinuria (n = 119)

^a ^not adjusted for calcium

^b ^not adjusted for phosphate

^c ^not adjusted for PTH

^d ^not adjusted for vitamin D

^e ^not adjusted for Klotho

^f^not adjusted for FGF23

^g^not adjusted for calcium and phosphate

^h^not adjusted for Klotho and FGF23.

CI: confidence interval; eGFR: estimated Glomerular Filtration Rate.

FGF23: Fibroblast growth factor 23; HTA: hypertension; PTH: parathyroid hormone; T_50_: Calcification propensity.

Ln: logarithmic transformation.

In summary, increased PTH and FGF23 and decreased 25D, Klotho and T_50_ were associated with IF. Although some of these markers were not independent predictors of the degree of fibrosis after adjustment, we attempted to determine their utility as markers of different stages of fibrosis. We therefore evaluated their discriminative performance in predicting low (≤20%) and significant (>40%) fibrosis. We built receiver operating characteristic (ROC) curves and reported area under the curve (AUC).

**Fig 2:**
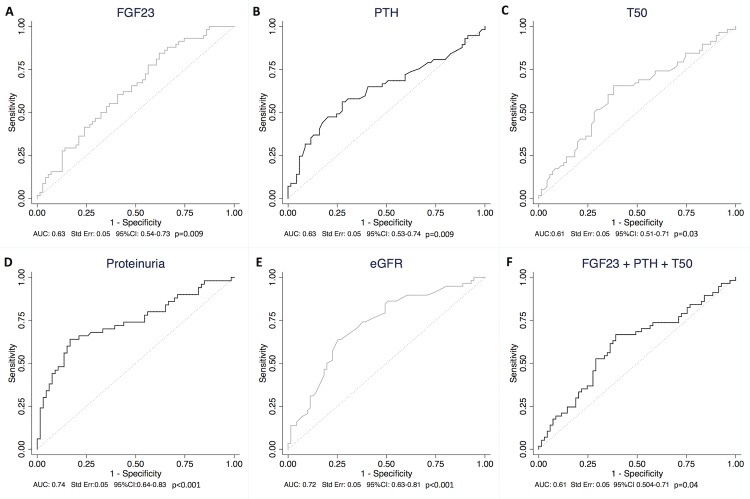
ROC curves of ln FGF23, ln PTH, T_50_, proteinuria and eGFR in predicting fibrosis ≤20%. ROC curve analysis of ln FGF23 (Fig 2A), ln PTH (Fig 2B), T_50_ (Fig 2C), proteinuria (Fig 2D), eGFR (Fig 2E) for the prediction of fibrosis ≤20%. (2F): ROC cuvre analysis of ln FGF23, ln PTH and T_50_ combined for the prediction of fibrosis ≤20%. As T_50_ and eGFR are markers that are negatively associated with fibrosis, we used the opposite values of those markers. AUC: Area Under the Curve; eGFR: estimated Glomerular Filtration Rate; FGF23: Fibroblast growth factor 23; Ln: log-transformed; PTH: parathyroid hormone; ROC: Receiver Operating Characteristic; T_50_: Calcification propensity.

Analysis revealed that FGF23, PTH and T_50_ were predictors of fibrosis ≤20%. The AUC was 0.63 for FGF23, 0.63 for PTH and 0.61 for T_50_ (Fig 2A, 2B and 2C). The ROC curve combining PTH and FGF23 for the prediction of fibrosis ≤20% only slightly improved prediction (AUC 0.66, Std Err: 0.05, 95%CI 0.56–0.75, p = 0.002). The ROC curve combining PTH, FGF23 and T_50_ did not improve the prediction of fibrosis ≤20% (Fig 2F). By comparison, AUC was 0.74 for proteinuria and 0.72 for eGFR (Fig 2D–2E). ROC curve analysis of eGFR and proteinuria combined did not improve AUC (AUC: 0.72, Std Err: 0.05, 95%CI: 0.63–0.0.82, p<0.001). Similarly, the ROC curve combining mineral markers (PTH, FGF23 and T_50_), proteinuria and eGFR did not improve the prediction of fibrosis ≤20% compared to eGFr only (AUC: 0.64, Std Err: 0.05, 95%CI: 0.54–0.74, p = 0.009).

25D, calcium, phosphate and Klotho were not significant predictors of low IF (S5 Fig).

**Fig 3:**
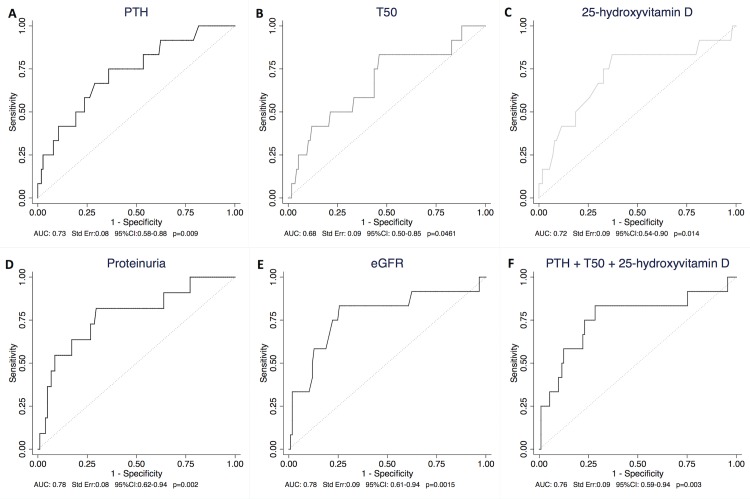
ROC curves of T_50_, 25D, PTH, proteinuria and eGFR in predicting Fibrosis >40%. As T_50_, vitamin D and eGFR are markers that are negatively associated with fibrosis, we used the opposite values of those markers. (**3A-B-C-D-E)** Separate ROC curves for ln PTH, T_50_, 25D, proteinuria and eGFR (**3F**) ROC curve for ln PTH, 25D and T_50_ combined. 25D: 25-hydroxyvitamin D; AUC: Area Under the Curve; eGFR: estimated Glomerular Filtration Rate; Ln: log-transformed; PTH: parathyroid hormone; ROC: Receiver Operating Characteristic; T_50_: Calcification propensity.

PTH, T_50_ and 25D were predictors of fibrosis >40% with AUC values of 0.73 for PTH, 0.68 for T_50_ and 0.72 for 25D (Fig 3A, 3B and 3C). The ROC curve combining PTH, 25D and T_50_ for the prediction of fibrosis >40% led to slightly better results (AUC 0.76) (Fig 3F). The AUC was not different from eGFR alone (AUC 0.78) (Fig 3E). The ROC curve combining mineral markers (PTH, T_50_ and 25D), proteinuria and eGFR did not improve the prediction of fibrosis >40% compared to eGFR alone (AUC: 0.76, Std Err: 0.09, 95%CI: 0.57–0.95, p = 0.004).

FGF23, calcium phosphate and Klotho were not significant predictors of extensive fibrosis (S6 Fig).

### Association of phosphocalcic biomarkers and T_50_ with chronic vascular lesions

**Fig 4:**
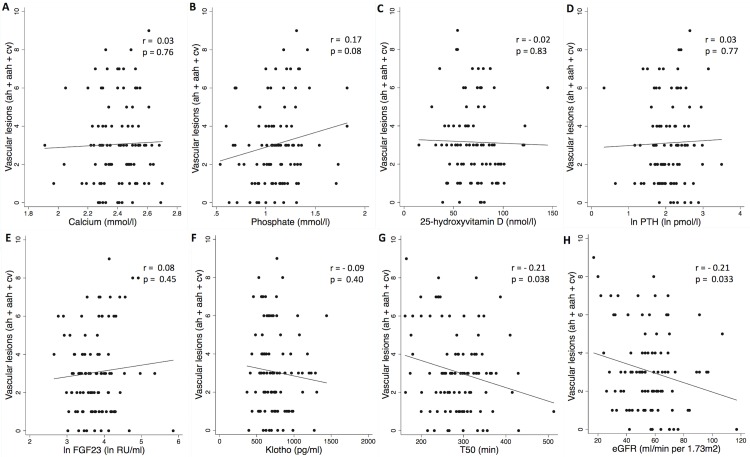
Phosphocalcic biomarkers and T_50_ associate with chronic vascular lesions as assessed by banff (ah+aah+cv) in renal allograft recipients. Scatter plot graphs of (A) calcium, (B) phosphate, (C) vitamin D, (D) ln PTH, (E) ln FGF23, (F) Klotho, (G) T_50,_ (H) eGFR versus vascular lesions. Each symbol represents one patient. The continuous line indicates least-square linear regression. Ah: arteriolar hyaline thickening; aah: circumferential hyaline arteriolar thickening; cv: vascular fibrous intimal thickening; eGFR: estimated Glomerular Filtration Rate; Ln: log-transformed; PTH: parathyroid hormone; T_50_: Calcification propensity.

Fig 4 displays the linear association of calcium, phosphate, 25D, PTH, FGF23, Klotho and T_50_ with Banff evaluation of chronic vascular lesions (ah+aah+cv) and their correlation coefficients. Only T_50_, eGFR and proteinuria were significantly associated to advancing vascular lesion (r = -0.21, p = 0.038 for T_50_, 0.21 for eGFR and 0.33 for proteinuria) (Fig 4G–4H and S2 Fig).

In the multivariate linear regression analysis, the significance of the association between T_50_ and vascular lesions was attenuated after adjusting for PTH and vitamin D (p = 0.051) but remained significant after adjustement for co-morbidities and eGFR (p = 0.048) and remained borderline after adjusting for proteinuria (p = 0.062) (Table 4).

**Table 4:** Associations of T_50_ with vascular lesions in kidney allograft recipients (n = 129), univariate and multivariate linear regression analysis.

Biomarkers	Coefficient	95% CI	p Value
**T**_**50**_ **(min)**			
Unadjusted	-0.007	-0.014 to 0.0004	0.038
Model 1	-0.007	-0.014 to 0.00003	0.051
Model 3	-0.006	-0.012 to -0.00004	0.048
Model 4	-0.006	-0.012 to 0.0003	0.062

Model 1: adjusted for ln PTH and vitamin D; Model 3: adjusted for model 1 and diabetes, HTA, graft vintage, eGFR; Model 4: adjusted for model 3 and proteinuria (n = 119); CI: confidence interval; eGFR: estimated Glomerular Filtration Rate was calculated according to the Chronic Kidney Disease Epidemiology Collaboration equation.

HTA: hypertension; PTH: parathyroid hormone; T_50_: Calcification propensity.

**Fig 5:**
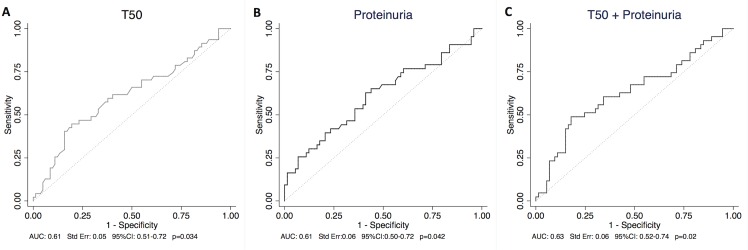
Vascular lesions estimated by BANFF: ROC curve of (5A) T_50_ and (5B) proteinuria in predicting significant vascular lesions (BANFF cv+ah+aah >5). (5C) ROC curve of T_50_ and proteinuria combined. Ah: arteriolar hyaline thickening; aah: circumferential hyaline arteriolar thickening; AUC: Area Under the Curve; cv: vascular fibrous intimal thickening; ROC: Receiver Operating Characteristic; T_50_: Calcification propensity.

We further evaluated the discriminative performance of T_50_ in predicting significant vascular lesions defined with a BANFF score for cv+ah+aah greater than 5. T_50_ and proteinuria were the only predictors of significant vascular lesion with an AUC of 0.61 for both (Fig 5A and 5B), and the combination of the two measures slightly enhanced discrimination (Fig 5C). Calcium, phosphate, PTH, 25D, FGF23, Klotho and eGFR were not significant predictors of vascular lesions (S7 Fig).

### Interstitial calcifications and phosphocalcic parameters

Von Kossa stainings were performed on biopsies available for additional coloration (n = 88) to indirectly localize mineralized areas in kidney tissue. Von Kossa stain positive deposits were observed within the cytoplasm of tubular epithelial cells, tubular lumen and interstitium in 8 biopsies (S8 Fig). There were no differences in the mean of all biomarkers between patients with and without calcified deposits (S1 Table).

## Discussion

In this retrospective study, we examined the association and predictive value of phosphocalcic markers and T_50_ with chronic renal histological lesions (IF and vascular lesions) in kidney allograft recipients. Our analysis revealed that PTH, 25D and T_50_ levels are associated to IF independently of renal function. When considered as biomarkers for IF <20 or >40%, mineral metabolism markers were however neither more discriminant, nor additive when compared to renal function parameters (eGFR and proteinuria). Despite the inferior performance as a marker of IF, the determinants of mineral metabolism assessed here might be of interest for therapy guidance. T_50_ and proteinuria were the only predictors of vascular lesions.

In our subset of kidney allograft recipients, similary to previous studies[26–28], we observed a good correlation between FGF23, Klotho and renal function as well as between renal function and IF. We confirmed a decrease in soluble Klotho and increase in FGF23 with declining eGFR. The associations were however low with structural lesions: FGF23 was positively correlated whereas Klotho negatively correlated with IF, but the strength of the association was only modest (p = 0.045) and disappeared after adjustement for other mineral metabolism, renal function and proteinuria. In contrast, PTH and 25D showed greater and independent associations with IF in our patient population, even after correction for renal function and proteinuria.

The physiopathological mechanism of the observed association of PTH and 25D to IF is not fully identified and the association observed does not imply a causal mechanism. Several hypotheses may be raised: PTH may participate in aggravating kidney fibrosis directly by an unknown mechanism, or PTH elevation may reflect an other unidentified third mediator linked to fibrosis. Of note, PTH was still associated to fibrosis after correction for vitamin D levels. Similarly, the role of vitamin D in several processes, either as a causal factor or as a biomarker indicative of a pathological state, is very much debated. Indeed, several studies indicate a relationship between hypovitaminosis D and survival, vascular calcification or inflammation [45]. However, the benefit of replacing native vitamin D is still to be demonstrated [46, 47]. Low Vitamin D levels may also indicate low levels of 1.25-dihydroxyvitamin D, although this was not measured in our patients [48]. Indeed, 1.25-dihydroxyvitamin D levels may be associated to fibrosis pathogenesis [49–51]. Low vitamin D may be a reflection of higher proteinuria. The observed association between vitamin D and fibrosis however persisted after adjustment for proteinuria. Low vitamin D may also be regarded as a marker of poor tubular endocytosis function, in line with the observation that small molecular weight protein in urine (including vitamin D binding protein) may be indicative of fibrosis [52, 53]. Regardless of the underlying mechanism, PTH and 25D levels were associated to IF, independently of renal function.

T_50_ is a novel biomarker predicting risk of cardiovascular events and all-cause mortality in patients with CKD. A high calcification propensity (i.e. low T_50_) is associated with increased all-cause mortality in a long-term follow-up [42, 44]. Interestingly low T_50_ is also predictive of worse evolution of renal function in kidney allograft recipients and one may hypothesize that the test might indicate chronic interstitial or vascular lesions. The association between T_50_ and IF was statistically significant, although not independent of proteinuria in our population, probably due to a lack of power. Another explanation maybe that T_50_ and proteinuria share some common pathophysiological pathways [44]. The correlation between proteinuria and T_50_ was however weak in our study.

We further examined the value of phophocalcic parameters in identifying different levels of fibrosis. Neither FGF23 nor Klotho were useful as predictors of advanced fibrosis. PTH, 25D and T_50_ showed good predictive value for advanced fibrosis identification, which increased when combining all measurements. Although each marker showed a lower independent predictive value compared to renal function as assessed by creatinine or eGFR, when combined the predictive value reached that of renal function. The addition of these markers to renal function did however not improve the prediction as assessed by the ROC curve. This suggests that PTH, T_50_ and 25D may be used as markers of fibrosis in condition where eGFR or creatinine may not be reliable, but do not have additional discrimination value in relation to eGFR. Conversely, low FGF23, PTH and T_50_ values may be useful in identifying patients with low fibrosis level (≤20%), although the obtained AUC was not very strong (AUC 0.63 and 0.61). When combining FGF23 and PTH, the AUC increased, but without improvement over eGFR. A low PTH and FGF23 level may thus identify patients without significant fibrosis when eGFR is not reliable, but does not add to eGFR prediction in our population.

The detection of vascular renal lesions in CKD patients is complex, since it is asymptomatic in early stages of CKD, and requires renal biopsy material for diagnosis. Therefore, methods aimed at noninvasive monitoring for early signs of vascular lesions are of clinical importance. In this study, the major parameters of mineral metabolism and vascular lesions were not associated with statistical significance. This is particularly surprising and disappointing in the case of Klotho. However, T_50_ levels continuously declined with vascular lesion. This association was attenuated after adjustment for proteinuria. When considered as biomarkers, only T_50_ and proteinuria were discriminative for significant vascular lesions, and even eGFR and creatinine were not. Since proteinuria is difficult to use as a marker of vascular lesion, T50 may have some clinical use for this purpose in the future. The associations between T_50_ and chronic interstitial lesions, either IF or vascular, is of interest and in fact, the T_50_ test monitors serum propensity to calcify [41]. Its association with, and predictive value for chronic histological lesions independently of renal function are surprising and in line with its predictive value for renal function decline [44]. This suggests that the pathophysiology of fibrosis and vascular lesions in the kidney may be dependent on the ability of the serum to calcify, and also indicates that this marker may be of use in combined scoring and potentially therapy guidance in the future. Indeed, it may be one of the only markers for vascular lesions, which may explain its predictive ability for CKD progression in kidney allograft recipients [44].

In this study, we were able to identify some predictors of chronic lesions. We may however lack enough power to identify other independent markers. We adjusted for the main confounders but residual confounding bias can still be present. Another limitation of our observations is that we studied kidney allograft recipients. Our findings can therefore not be extrapolated to native kidneys. Indeed, the pathophysiology of fibrosis and vascular lesions in allograft recipients may be less dependent on mineral metabolism adaptations than in native patients, explaining the low predictive value of FGF23 and Klotho for fibrosis and vascular lesions. These markers may be more efficient in identifying vascular lesions and fibrosis in native kidney disease patients, although this remains to be tested. Importantly, vascular lesions may be more dependent on medication such as calcineurin inhibitors or steroids in kidney transplanted patients, than on mineral metabolism adaptations.

In summary, we observe that in kidney allograft recipients, some mineral metabolism markers are associated to chronic histological lesions independently of eGFR. However, when considered as biomarkers of severity of chronic lesions, they do not show superiority or additive value to renal function assesment by eGFR, proteinuria or creatinine values. Only T_50_ appears to have a more specific predictive value for vascular lesions, and therefore allograft prognosis. This may be related to the specific pathophysiology of fibrosis in kidney allograft patients.

## Materials and Methods

### Patients

We designed a retrospective study including adult kidney allograft recipients having a kidney biopsy. Patients 18 years of age or older, who received a kidney transplant between 1982 and 2013 and who were followed routinely in 2015 in Nephrology at the University Hospital of Geneva, were eligible for enrollment. Two nephrologists, not in charge of the patients, randomly selected 150 patients having undergone biopsy between January 2007 to December 2014. If a patient had more than one biopsy, we selected the last one available. On average, 80 to 90 biopsies are performed per year in our center. We excluded 21 patients because of the lack of available serum at the time of biopsy leaving 129 patients for the present analysis.

The indications for biopsies were protocol biopsies at 1, 5, 10 or 20 years after transplantation (n = 115), or clinically-driven biopsies (n = 14) (Table 2). Clinical indications were a rise in serum creatinine (n = 6), proteinuria (n = 4), both (n = 3) or rise of donor specific antibodies (n = 1). In all kidney transplant patients, additional serum was collected yearly and stored at -80°C as part of our local transplant routine practice. We retrieved the serum taken closest to the time of the biopsy (with a maximum of 4 months distance from the biopsy and usually at the time of biopsy).

The study was approved by the local ethical committee for human studies of Geneva, Switzerland (Commission Cantonale d’Ethique de la recherche, CCER, directed by Pr. Bernard Hirschel) and performed according to the Declaration of Helsinki principles. All the patients were contacted to provide written informed consent to participate in this retrospective study. None of the transplant donors were from a vulnerable population and all donors or next of kin provided written informed consent that was freely given.

### Baseline investigation and laboratory measurements

Baseline characteristics, including medical history, co-morbidities and treatment, were collected through patient records. Patient’s blood pressure, weight and size were measured routinely during follow-up visits.

Serum creatinine and other standard laboratory values were measured during routine follow-up visits or hospitalizations and recorded at the time of collected sample. Standard biochemical analyses were performed in Geneva University Hospital Laboratory using the routine automated analyzers. The eGFR was calculated using the Chronic Kidney Disease Epidemiology Collaboration equation (CKD-EPI) [54]. Creatinine was measured by Jaffé-kinetics using IDMS-traceable methods.

Frozen samples were used for measurements of FGF23, Klotho and T_50_ in batch. Serum FGF23 levels were measured by the ELISA system using the C-TER Immunotopics kit [55]. An Elisa from IBL was used for serum levels measurement of soluble Klotho [56]. The serum calcification propensity test (T_50_) was performed using a Nephelostar nephelometer in University Hospital Bern, Switzerland [41]. Technicians from the laboratories were blinded to the clinical data and other results.

Renal fibrosis was assessed in the kidney biopsy using two distinct methods. Masson trichrome stained kidney sections were assessed quantitatively by two expert pathologists (S.M and O.S) blinded to the other results. The severity of renal fibrosis was scored subjectively from 0 to 100% for each patient. Furthermore, renal fibrosis was quantified using the BANFF criteria: ci (interstitial fibrosis) and ct (tubular atrophy) with a minimal of 0 and maximal of 6. Due to a good correlation between the two methods (r = 0.9; p <0.001), we used renal fibrosis as a continuous variable (0 to 100%) for all analyses. Analysis of vascular lesion was determined using BANFF criteria from the pathologist report: ah (arteriolar hyaline thickening), aah (circumferential hyaline arteriolar thickening) and cv (vascular fibrous intimal thickening) with a minimal of 0 and maximal of 9. Samples available were n = 121 for ah, n = 119 for aah and n = 118 for cv, depending on the presence of vessels on the biopsy. To complete our examination, Von Kossa stainings were performed on biopsies available for supplementary coloration to indirectly localize mineralized areas in kidney tissues. Von Kossa stain deposits were blindedly analyzed by one nephrologist and one pathologist. We divided patients in two groups depending on results (postitive or negative Von Kossa Staining).

### Statistical analysis

Continuous variables are expressed as mean ± standard deviation or median and interquartile range according to the distribution. Categorical variables are expressed as numbers and percentages. Normality was tested using the Shapiro–Francia test for normal data. FGF23, PTH and proteinuria values were logarithmically transformed on a natural logarithm before analyses due to abnormal distribution. Because of non-normality of the distribution, the Mann-Whitney U-test was used for comparing values of phosphocalcic biomarkers and T_50_ between the two different groups of Von Kossa. For simple correlation analyses between phosphocalcic parameters, including T_50_, and histological parameters, we performed Pearson’s tests after controlling the linear associations with scatter plots. We conducted univariate and multivariate linear regression for continuous outcomes such as the percentage of fibrosis and vascular lesions. We created different models including our variable of interest from the phosphocalcic metabolism: calcium, phosphate, PTH, vitamin D, FGF23, Klotho and T_50_. Models were first adjusted for other phosphocalcic parameters (model 1), secondly for FGF23 and Klotho (model 2), followed by diabetes, hypertension, graft vintage, eGFR (model 3) and finally proteinuria (model 4).

We then stratified fibrosis and vascular lesions into categories. We tested IF at two different levels (≤20% and >40%). Vascular lesions were obtained by summing the determinant of vascular component of BANFF score (cv, ah and aah). Significant vascular lesions were defined as those having a score >5. To evaluate the discriminative performance of those markers to predict different levels of fibrosis and vascular lesions, we constructed receiver operating characteristic (ROC) curves. For markers inversely associated with fibrosis, we used their opposite values to assess their diagnostic performance for the different fibrosis levels or the vascular cut-off and obtaining ROC curves above the identity line. We reported AUC values with 95%CI to compare prognostic value of each parameter.

Statistical analyses were performed using STATA 13.1 (StataCorp, College Station, TX, USA). Two-sided values of p<0.05 were considered statistically significant.

## Supporting Information

S1 Fig Correlations between phosphocalcic biomarkers, T_50,_ proteinuria and eGFR in renal allograft recipients (n = 129). Scatter plot graphs of (A) calcium, (B) phosphate, (C) vitamin D, (D) lnPTH, (E) lnFGF23, (F) Klotho, (G) T50, (H) proteinuria versus eGFR. Each symbol represents one patient. The continuous lines indicates least-square linear regression. eGFR: estimated Glomerular Filtration Rate; FGF23: Fibroblast growth factor 23; PTH: parathyroid hormone; T_50_: Calcification propensity; Ln: log-transformed.
(TIF)

S2 Fig **Correlations between (A) creatinine and (B) proteinuria and fibrosis(%). Correlation between (C) creatinine and (D) proteinuria and chronic vascular lesions as assessed by banff (ah+aah+cv) in renal allograft recipients.** Each symbol represents one patient. The continuous lines indicates least-square linear regression. Ln: log-transformed.
(TIF)

S3 Fig **Boxplot of (A) calcium, (B) phosphate, (C)vitamin D, (D) ln PTH, (E) ln FGF23, (F) Klotho, (G) T50, (H) eGFR, (I) creatinine and (J) ln proteinuria by category of fibrosis as assess by BANFF classification (0–25%; 26–50% >50% of fibrosis).** The lower border of the box and the upper border of the box represent the first quartile and third quartile, respectively. The whiskers indicate the 1st and 99th percentiles. eGFR: estimated Glomerular Filtration Rate; FGF23: Fibroblast growth factor 23; PTH: parathyroid hormone; T_50_: Calcification propensity; Ln: log-transformed.
(TIF)

S4 Fig Correlations between phosphocalcic biomarkers, T_50,_ proteinuria, creatinine, eGFR and fibrosis (%) in renal allograft recipients (n = 115). Scatter plot graphs of (A) calcium, (B) phosphate, (C) vitamin D, (D) lnPTH, (E) lnFGF23, (F) Klotho, (G) T50, (H) eGFR, (I) creatinine, (J) ln proteinuria versus fibrosis (%). Each symbol represents one patient. The continuous lines indicates least-square linear regression. eGFR: estimated Glomerular Filtration Rate; FGF23: Fibroblast growth factor 23; PTH: parathyroid hormone; T_50_: Calcification propensity; Ln: log-transformed.
(TIF)

S5 Fig ROC curves of creatinine, vitamin D, calcium, phosphate and Klotho in predicting fibrosis ≤20%. ROC curve analysis of creatinine (S5A Fig), vitamin D (S5B Fig), calcium (S5C Fig), phosphate (S5D Fig) and 5Klotho (S5E Fig) for the prediction of fibrosis ≤20%. As vitamin D and Klotho are markers that are negatively associated with fibrosis, we used the opposite values of those markers. AUC: Area Under the Curve; ROC: Receiver Operating Characteristic.
(TIF)

S6 Fig ROC curves of creatinine, vitamin D, calcium, phosphate and Klotho in predicting fibrosis >40%. ROC curve analysis of creatinine (S6A Fig), vitamin D (S6B Fig), calcium (S6C Fig), phosphate (S6D Fig) and Klotho (S6E Fig) for the prediction of fibrosis >40%. As vitamin D and Klotho are markers that are negatively associated with fibrosis, we used the opposite values of those markers. AUC: Area Under the Curve; ROC: Receiver Operating Characteristic.
(TIF)

S7 Fig **Vascular lesions estimated by BANFF: ROC curves of calcium (A), phosphate (B), PTH (C), vitamin D (D), FGF23 (E), Klotho (F), eGFR (G) and creatinine (H) in predicting significant vascular lesions (BANFF cv+ah+aah >5).** Ah: arteriolar hyaline thickening; aah: circumferential hyaline arteriolar thickening; AUC: Area Under the Curve; cv: vascular fibrous intimal thickening; FGF23: Fibroblast growth factor 23; PTH: parathyroid hormone; ROC: Receiver Operating Characteristic.
(TIF)

S8 Fig Example of Von Kossa stain positive deposits in a kidney biopsy. (TIF)

S1 Table Level value of phosphocalcic biomarkers and T_50_ across Von Kossa results (n = 88). (PDF)

## References

[1] Schainuck LI, Striker GE, Cutler RE, Benditt EP. Structural-functional correlations in renal disease. II. The correlations. Human pathology. 1970;1(4):631–41. 5521736 10.1016/s0046-8177(70)80061-2

[2] Cosio FG, Grande JP, Larson TS, Gloor JM, Velosa JA, Textor SC, et al Kidney allograft fibrosis and atrophy early after living donor transplantation. American journal of transplantation: official journal of the American Society of Transplantation and the American Society of Transplant Surgeons. 2005;5(5):1130–6. 10.1111/j.1600-6143.2005.00811.x 15816896

[3] Park WD, Griffin MD, Cornell LD, Cosio FG, Stegall MD. Fibrosis with inflammation at one year predicts transplant functional decline. Journal of the American Society of Nephrology: JASN. 2010;21(11):1987–97. PubMed Central PMCID: PMC3014013. 10.1681/ASN.2010010049 20813870 PMC3014013

[4] Banfi G, Bertani T, Boeri V, Faraggiana T, Mazzucco G, Monga G, et al Renal vascular lesions as a marker of poor prognosis in patients with lupus nephritis. Gruppo Italiano per lo Studio della Nefrite Lupica (GISNEL). American journal of kidney diseases: the official journal of the National Kidney Foundation. 1991;18(2):240–8. 1867181 10.1016/s0272-6386(12)80885-7

[5] Marques ID, Repizo LP, Pontelli R, de Paula FJ, Nahas WC, David DS, et al [Vasculopathy in the kidney allograft at time of transplantation delays recovery of graft function after deceased-donor kidney transplantation]. J Bras Nefrol. 2014;36(1):54–8. 24676615 10.5935/0101-2800.20140010

[6] Hernandez D, Rufino M, Bartolomei S, Gonzalez-Rinne A, Lorenzo V, Cobo M, et al Clinical impact of preexisting vascular calcifications on mortality after renal transplantation. Kidney international. 2005;67(5):2015–20. 10.1111/j.1523-1755.2005.00303.x 15840052

[7] Cosio FG, Grande JP, Wadei H, Larson TS, Griffin MD, Stegall MD. Predicting subsequent decline in kidney allograft function from early surveillance biopsies. American journal of transplantation: official journal of the American Society of Transplantation and the American Society of Transplant Surgeons. 2005;5(10):2464–72. 10.1111/j.1600-6143.2005.01050.x 16162196

[8] Grimm PC, Nickerson P, Gough J, McKenna R, Jeffery J, Birk P, et al Quantitation of allograft fibrosis and chronic allograft nephropathy. Pediatric transplantation. 1999;3(4):257–70. 10562970 10.1034/j.1399-3046.1999.00044.x

[9] Risdon RA, Sloper JC, De Wardener HE. Relationship between renal function and histological changes found in renal-biopsy specimens from patients with persistent glomerular nephritis. Lancet. 1968;2(7564):363–6. 4173786 10.1016/s0140-6736(68)90589-8

[10] Working Group of the International Ig ANN, the Renal Pathology S, Cattran DC, Coppo R, Cook HT, Feehally J, et al The Oxford classification of IgA nephropathy: rationale, clinicopathological correlations, and classification. Kidney international. 2009;76(5):534–45. 10.1038/ki.2009.243 19571791

[11] Whittier WL, Korbet SM. Timing of complications in percutaneous renal biopsy. Journal of the American Society of Nephrology: JASN. 2004;15(1):142–7. 14694166 10.1097/01.asn.0000102472.37947.14

[12] Kuro-o M, Matsumura Y, Aizawa H, Kawaguchi H, Suga T, Utsugi T, et al Mutation of the mouse klotho gene leads to a syndrome resembling ageing. Nature. 1997;390(6655):45–51. 10.1038/36285 9363890

[13] Hu MC, Kuro-o M, Moe OW. Renal and extrarenal actions of Klotho. Semin Nephrol. 2013;33(2):118–29. PubMed Central PMCID: PMCPMC3593734. 10.1016/j.semnephrol.2012.12.013 23465499 PMC3593734

[14] Pavik I, Jaeger P, Ebner L, Wagner CA, Petzold K, Spichtig D, et al Secreted Klotho and FGF23 in chronic kidney disease Stage 1 to 5: a sequence suggested from a cross-sectional study. Nephrology, dialysis, transplantation: official publication of the European Dialysis and Transplant Association—European Renal Association. 2013;28(2):352–9. 10.1093/ndt/gfs460 23129826

[15] Semba RD, Cappola AR, Sun K, Bandinelli S, Dalal M, Crasto C, et al Plasma klotho and cardiovascular disease in adults. J Am Geriatr Soc. 2011;59(9):1596–601. PubMed Central PMCID: PMCPMC3486641. 10.1111/j.1532-5415.2011.03558.x 21883107 PMC3486641

[16] Zhou L, Li Y, Zhou D, Tan RJ, Liu Y. Loss of Klotho contributes to kidney injury by derepression of Wnt/beta-catenin signaling. Journal of the American Society of Nephrology: JASN. 2013;24(5):771–85. PubMed Central PMCID: PMCPMC3636797. 10.1681/ASN.2012080865 23559584 PMC3636797

[17] Hu MC, Shiizaki K, Kuro-o M, Moe OW. Fibroblast growth factor 23 and Klotho: physiology and pathophysiology of an endocrine network of mineral metabolism. Annu Rev Physiol. 2013;75:503–33. PubMed Central PMCID: PMCPMC3770142. 10.1146/annurev-physiol-030212-183727 23398153 PMC3770142

[18] Kim HR, Nam BY, Kim DW, Kang MW, Han JH, Lee MJ, et al Circulating alpha-klotho levels in CKD and relationship to progression. American journal of kidney diseases: the official journal of the National Kidney Foundation. 2013;61(6):899–909. 23540260 10.1053/j.ajkd.2013.01.024

[19] Kitagawa M, Sugiyama H, Morinaga H, Inoue T, Takiue K, Ogawa A, et al A decreased level of serum soluble Klotho is an independent biomarker associated with arterial stiffness in patients with chronic kidney disease. PloS one. 2013;8(2):e56695 PubMed Central PMCID: PMCPMC3576368. 10.1371/journal.pone.0056695 23431388 PMC3576368

[20] Hu MC, Shi M, Zhang J, Quinones H, Griffith C, Kuro-o M, et al Klotho deficiency causes vascular calcification in chronic kidney disease. Journal of the American Society of Nephrology: JASN. 2011;22(1):124–36. PubMed Central PMCID: PMCPMC3014041. 10.1681/ASN.2009121311 21115613 PMC3014041

[21] Fliser D, Kollerits B, Neyer U, Ankerst DP, Lhotta K, Lingenhel A, et al Fibroblast growth factor 23 (FGF23) predicts progression of chronic kidney disease: the Mild to Moderate Kidney Disease (MMKD) Study. Journal of the American Society of Nephrology: JASN. 2007;18(9):2600–8. 10.1681/ASN.2006080936 17656479

[22] Seiler S, Reichart B, Roth D, Seibert E, Fliser D, Heine GH. FGF-23 and future cardiovascular events in patients with chronic kidney disease before initiation of dialysis treatment. Nephrology, dialysis, transplantation: official publication of the European Dialysis and Transplant Association—European Renal Association. 2010;25(12):3983–9. 10.1093/ndt/gfq309 20525642

[23] Gutierrez OM, Mannstadt M, Isakova T, Rauh-Hain JA, Tamez H, Shah A, et al Fibroblast growth factor 23 and mortality among patients undergoing hemodialysis. N Engl J Med. 2008;359(6):584–92. PubMed Central PMCID: PMCPMC2890264. 10.1056/NEJMoa0706130 18687639 PMC2890264

[24] Gutierrez OM, Januzzi JL, Isakova T, Laliberte K, Smith K, Collerone G, et al Fibroblast growth factor 23 and left ventricular hypertrophy in chronic kidney disease. Circulation. 2009;119(19):2545–52. PubMed Central PMCID: PMCPMC2740903. 10.1161/CIRCULATIONAHA.108.844506 19414634 PMC2740903

[25] Mirza MA, Hansen T, Johansson L, Ahlstrom H, Larsson A, Lind L, et al Relationship between circulating FGF23 and total body atherosclerosis in the community. Nephrology, dialysis, transplantation: official publication of the European Dialysis and Transplant Association—European Renal Association. 2009;24(10):3125–31. 10.1093/ndt/gfp205 19429932

[26] Bleskestad IH, Thorsen IS, Jonsson G, Skadberg O, Bergrem H, Goransson LG. Soluble Klotho and intact fibroblast growth factor 23 in long-term kidney transplant patients. Eur J Endocrinol. 2015;172(4):343–50. 10.1530/EJE-14-0457 25572388

[27] Malyszko J, Koc-Zorawska E, Matuszkiewicz-Rowinska J, Malyszko J. FGF23 and Klotho in relation to markers of endothelial dysfunction in kidney transplant recipients. Transplantation proceedings. 2014;46(8):2647–50. 10.1016/j.transproceed.2014.09.015 25380886

[28] Sanchez Fructuoso AI, Maestro ML, Perez-Flores I, Valero R, Rafael S, Veganzones S, et al Serum level of fibroblast growth factor 23 in maintenance renal transplant patients. Nephrology, dialysis, transplantation: official publication of the European Dialysis and Transplant Association—European Renal Association. 2012;27(11):4227–35. 10.1093/ndt/gfs409 23144073

[29] Mehta R, Ying GS, Houston S, Isakova T, Nessel L, Ojo A, et al Phosphate, fibroblast growth factor 23 and retinopathy in chronic kidney disease: the Chronic Renal Insufficiency Cohort Study. Nephrology, dialysis, transplantation: official publication of the European Dialysis and Transplant Association—European Renal Association. 2015;30(9):1534–41. PubMed Central PMCID: PMCPMC4550441. 10.1093/ndt/gfv123 PMC4550441 25910495

[30] Wolf M, Molnar MZ, Amaral AP, Czira ME, Rudas A, Ujszaszi A, et al Elevated fibroblast growth factor 23 is a risk factor for kidney transplant loss and mortality. Journal of the American Society of Nephrology: JASN. 2011;22(5):956–66. PubMed Central PMCID: PMCPMC3083317. 10.1681/ASN.2010080894 21436289 PMC3083317

[31] Baia LC, Heilberg IP, Navis G, de Borst MH, investigators N. Phosphate and FGF-23 homeostasis after kidney transplantation. Nat Rev Nephrol. 2015;11(11):656–66. 10.1038/nrneph.2015.153 26416497

[32] Dhingra R, Sullivan LM, Fox CS, Wang TJ, D'Agostino RB Sr., Gaziano JM, et al Relations of serum phosphorus and calcium levels to the incidence of cardiovascular disease in the community. Arch Intern Med. 2007;167(9):879–85. 10.1001/archinte.167.9.879 17502528

[33] Kestenbaum B, Sampson JN, Rudser KD, Patterson DJ, Seliger SL, Young B, et al Serum phosphate levels and mortality risk among people with chronic kidney disease. J Am Soc Nephrol. 2005;16(2):520–8. 10.1681/ASN.2004070602 15615819

[34] Stevens KK, Patel RK, Mark PB, Delles C, Jardine AG. Phosphate as a cardiovascular risk factor: effects on vascular and endothelial function. Lancet. 2015;385 Suppl 1:S10. 10.1016/S0140-6736(15)60325-7 26312829

[35] Hiemstra TF, Brown AJ, Chaudhry AN, Walsh M. Association of calcium, phosphate and parathyroid hormone with renal allograft function: a retrospective cohort study. Am J Nephrol. 2013;37(4):339–45. 10.1159/000348376 23548209

[36] Kovesdy CP, Ahmadzadeh S, Anderson JE, Kalantar-Zadeh K. Secondary hyperparathyroidism is associated with higher mortality in men with moderate to severe chronic kidney disease. Kidney international. 2008;73(11):1296–302. 10.1038/ki.2008.64 18337714

[37] Tseng PY, Yang WC, Yang CY, Tarng DC. Long-term Outcomes of Parathyroidectomy in Kidney Transplant Recipients with Persistent Hyperparathyroidism. Kidney Blood Press Res. 2015;40(4):386–94. 10.1159/000368514 26184764

[38] Bleskestad IH, Bergrem H, Leivestad T, Hartmann A, Goransson LG. Parathyroid hormone and clinical outcome in kidney transplant patients with optimal transplant function. Clin Transplant. 2014;28(4):479–86. 10.1111/ctr.12341 25649861

[39] Pihlstrom H, Dahle DO, Mjoen G, Pilz S, Marz W, Abedini S, et al Increased risk of all-cause mortality and renal graft loss in stable renal transplant recipients with hyperparathyroidism. Transplantation. 2015;99(2):351–9. 10.1097/TP.0000000000000583 25594550

[40] Yilmaz MI, Sonmez A, Saglam M, Yaman H, Kilic S, Turker T, et al Longitudinal analysis of vascular function and biomarkers of metabolic bone disorders before and after renal transplantation. Am J Nephrol. 2013;37(2):126–34. 10.1159/000346711 23391995

[41] Pasch A, Farese S, Graber S, Wald J, Richtering W, Floege J, et al Nanoparticle-based test measures overall propensity for calcification in serum. Journal of the American Society of Nephrology: JASN. 2012;23(10):1744–52. PubMed Central PMCID: PMCPMC3458464. 10.1681/ASN.2012030240 22956818 PMC3458464

[42] Smith ER, Ford ML, Tomlinson LA, Bodenham E, McMahon LP, Farese S, et al Serum calcification propensity predicts all-cause mortality in predialysis CKD. J Am Soc Nephrol. 2014;25(2):339–48. PubMed Central PMCID: PMC3904573. 10.1681/ASN.2013060635 24179171 PMC3904573

[43] Dahle DO, Asberg A, Hartmann A, Holdaas H, Bachtler M, Jenssen TG, et al Serum Calcification Propensity Is a Strong and Independent Determinant of Cardiac and All-Cause Mortality in Kidney Transplant Recipients. American journal of transplantation: official journal of the American Society of Transplantation and the American Society of Transplant Surgeons. 2015. 10.1111/ajt.13443 26375609

[44] Keyzer CA, de Borst MH, van den Berg E, Jahnen-Dechent W, Arampatzis S, Farese S, et al Calcification Propensity and Survival among Renal Transplant Recipients. Journal of the American Society of Nephrology: JASN. 2015. 10.1681/ASN.2014070670 PMC4696561 25925688

[45] Lucisano S, Buemi M, Passantino A, Aloisi C, Cernaro V, Santoro D. New insights on the role of vitamin D in the progression of renal damage. Kidney Blood Press Res. 2013;37(6):667–78. 10.1159/000355747 24356557

[46] Zhang Q, Li M, Zhang T, Chen J. Effect of Vitamin D Receptor Activators on Glomerular Filtration Rate: A Meta-Analysis and Systematic Review. PloS one. 2016;11(1):e0147347 PubMed Central PMCID: PMCPMC4727919. 10.1371/journal.pone.0147347 26812502 PMC4727919

[47] Courbebaisse M, Xu-Dubois YC, Thervet E, Prie D, Zuber J, Kreis H, et al Cholecalciferol supplementation does not protect against renal allograft structural and functional deterioration: a retrospective study. Transplantation. 2011;91(2):207–12. 10.1097/TP.0b013e318200ba37 21131899

[48] Bosworth CR, Levin G, Robinson-Cohen C, Hoofnagle AN, Ruzinski J, Young B, et al The serum 24,25-dihydroxyvitamin D concentration, a marker of vitamin D catabolism, is reduced in chronic kidney disease. Kidney international. 2012;82(6):693–700. PubMed Central PMCID: PMCPMC3434313. 10.1038/ki.2012.193 22648296 PMC3434313

[49] Tan X, Li Y, Liu Y. Paricalcitol attenuates renal interstitial fibrosis in obstructive nephropathy. Journal of the American Society of Nephrology: JASN. 2006;17(12):3382–93. 10.1681/ASN.2006050520 17082242

[50] Zhang Z, Zhang Y, Ning G, Deb DK, Kong J, Li YC. Combination therapy with AT1 blocker and vitamin D analog markedly ameliorates diabetic nephropathy: blockade of compensatory renin increase. Proc Natl Acad Sci U S A. 2008;105(41):15896–901. PubMed Central PMCID: PMCPMC2562415. 10.1073/pnas.0803751105 18838678 PMC2562415

[51] Tan X, He W, Liu Y. Combination therapy with paricalcitol and trandolapril reduces renal fibrosis in obstructive nephropathy. Kidney international. 2009;76(12):1248–57. 10.1038/ki.2009.346 19759524 PMC5527548

[52] Mirkovic K, Doorenbos CR, Dam WA, Lambers Heerspink HJ, Slagman MC, Nauta FL, et al Urinary vitamin D binding protein: a potential novel marker of renal interstitial inflammation and fibrosis. PloS one. 2013;8(2):e55887 PubMed Central PMCID: PMCPMC3569442. 10.1371/journal.pone.0055887 23409077 PMC3569442

[53] Doorenbos CR, van den Born J, Navis G, de Borst MH. Possible renoprotection by vitamin D in chronic renal disease: beyond mineral metabolism. Nat Rev Nephrol. 2009;5(12):691–700. 10.1038/nrneph.2009.185 19859070

[54] Levey AS, Stevens LA, Schmid CH, Zhang YL, Castro AF 3rd, Feldman HI, et al A new equation to estimate glomerular filtration rate. Annals of internal medicine. 2009;150(9):604–12. PubMed Central PMCID: PMCPMC2763564. 19414839 10.7326/0003-4819-150-9-200905050-00006 PMC2763564

[55] Heijboer AC, Levitus M, Vervloet MG, Lips P, ter Wee PM, Dijstelbloem HM, et al Determination of fibroblast growth factor 23. Ann Clin Biochem. 2009;46(Pt 4):338–40. 10.1258/acb.2009.009066 19564163

[56] Pedersen L, Pedersen SM, Brasen CL, Rasmussen LM. Soluble serum Klotho levels in healthy subjects. Comparison of two different immunoassays. Clin Biochem. 2013;46(12):1079–83. 10.1016/j.clinbiochem.2013.05.046 23707222

