# Supplementary figures and images for: Phosphocalcic Markers and Calcification Propensity for Assessment of Interstitial Fibrosis and Vascular Lesions in Kidney Allograft Recipients
Source: PLoS One. 2016 Dec 30;11(12):e0167929. doi: 10.1371/journal.pone.0167929 (PMC5201285; doi:10.1371/journal.pone.0167929)

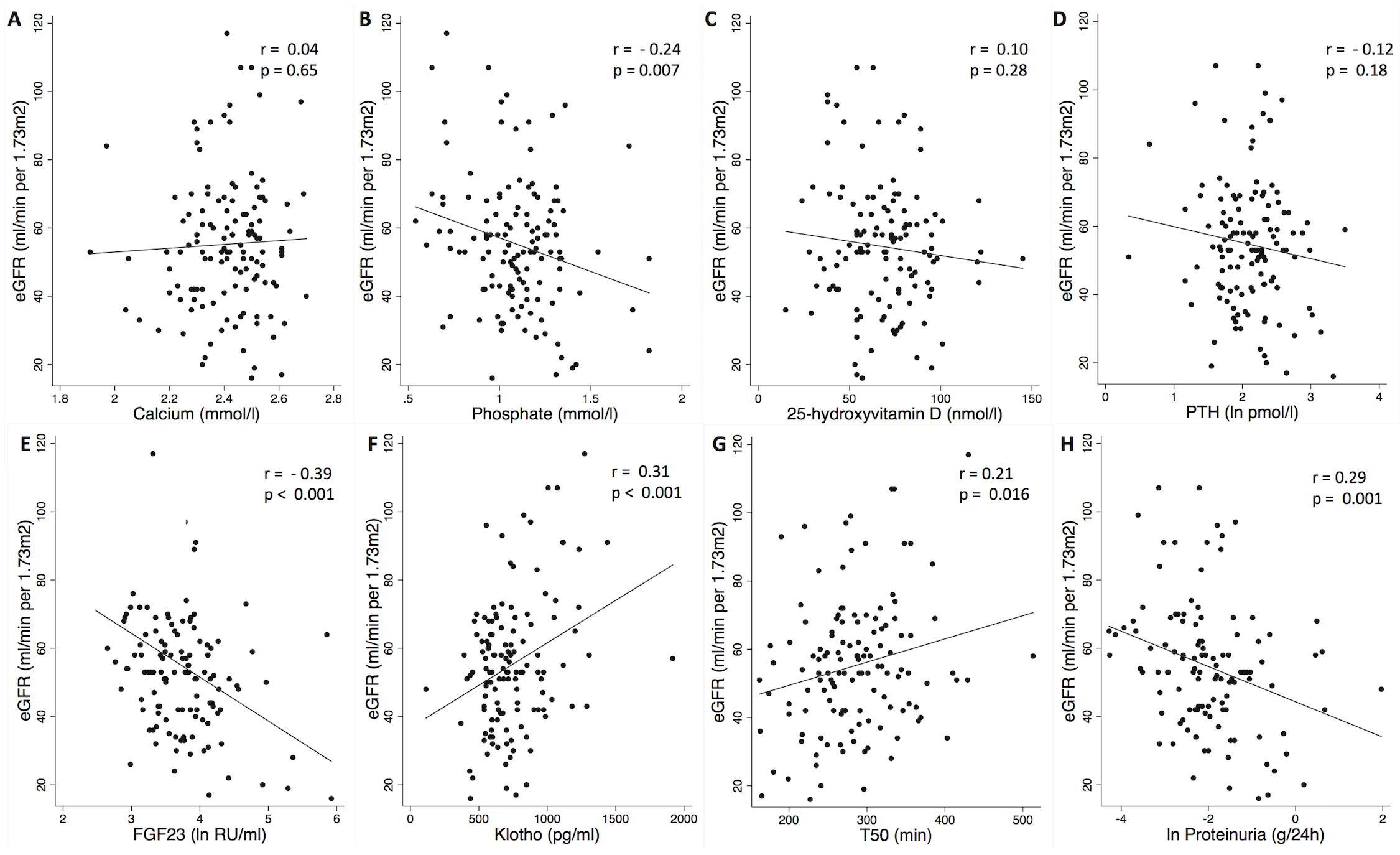

Supplement: S1 Fig — Scatter plot graphs of (A) calcium, (B) phosphate, (C) vitamin D, (D) lnPTH, (E) lnFGF23, (F) Klotho, (G) T50, (H) proteinuria versus eGFR. Each symbol represents one patient. The continuous lines indicates least-square linear regression. eGFR: estimated Glomerular Filtration Rate; FGF23: Fibroblast growth factor 23; PTH: parathyroid hormone; T50: Calcification propensity; Ln: log-transformed. (TIF) [file pone.0167929.s001.tif]

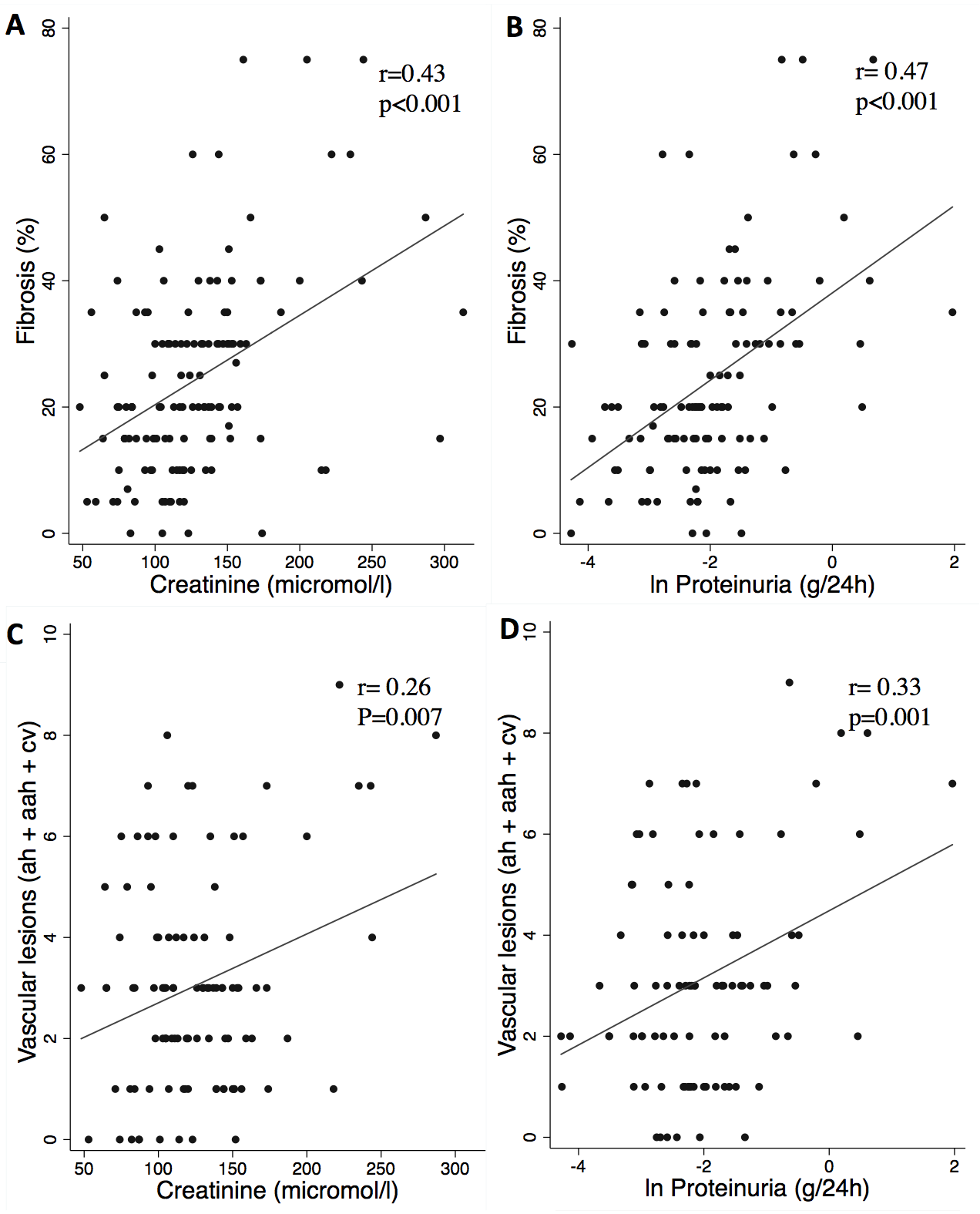

Supplement: S2 Fig — Correlations between (A) creatinine and (B) proteinuria and fibrosis(%). Correlation between (C) creatinine and (D) proteinuria and chronic vascular lesions as assessed by banff (ah+aah+cv) in renal allograft recipients. Each symbol represents one patient. The continuous lines indicates least-square linear regression. Ln: log-transformed. (TIF) [file pone.0167929.s002.tif]

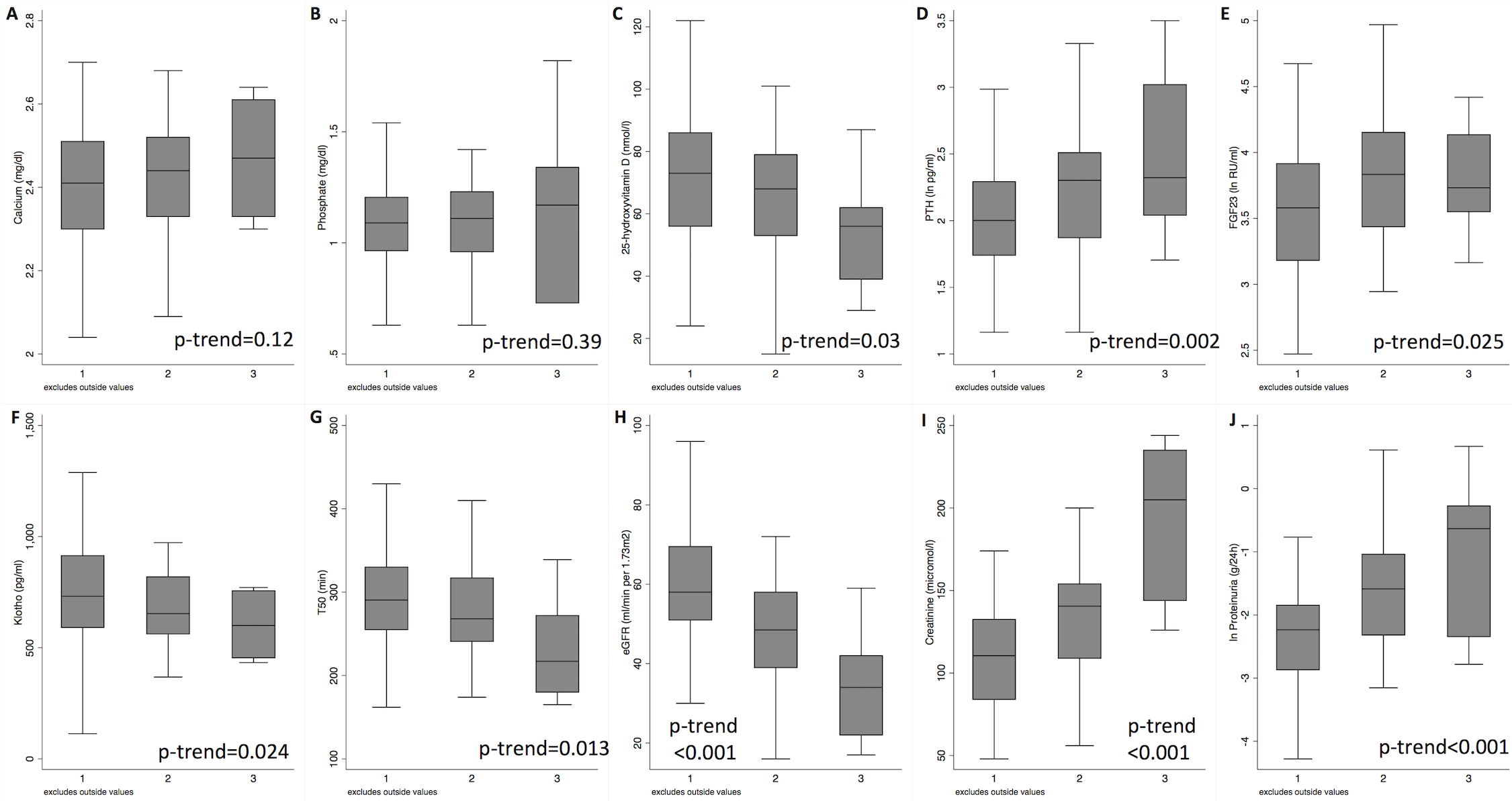

Supplement: S3 Fig — Boxplot of (A) calcium, (B) phosphate, (C)vitamin D, (D) ln PTH, (E) ln FGF23, (F) Klotho, (G) T50, (H) eGFR, (I) creatinine and (J) ln proteinuria by category of fibrosis as assess by BANFF classification (0–25%; 26–50% >50% of fibrosis). The lower border of the box and the upper border of the box represent the first quartile and third quartile, respectively. The whiskers indicate the 1st and 99th percentiles. eGFR: estimated Glomerular Filtration Rate; FGF23: Fibroblast growth factor 23; PTH: parathyroid hormone; T50: Calcification propensity; Ln: log-transformed. (TIF) [file pone.0167929.s003.tif]

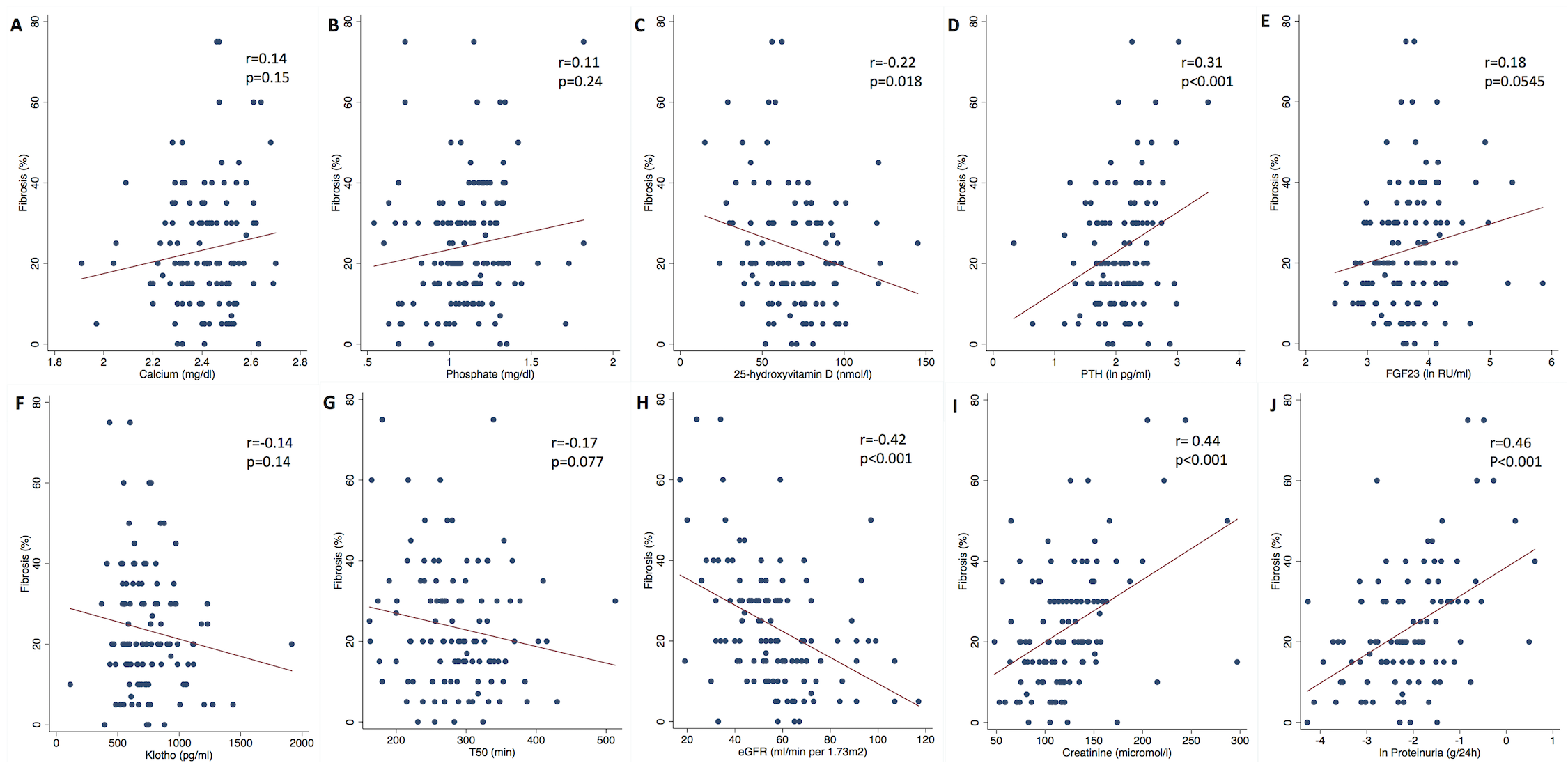

Supplement: S4 Fig — Scatter plot graphs of (A) calcium, (B) phosphate, (C) vitamin D, (D) lnPTH, (E) lnFGF23, (F) Klotho, (G) T50, (H) eGFR, (I) creatinine, (J) ln proteinuria versus fibrosis (%). Each symbol represents one patient. The continuous lines indicates least-square linear regression. eGFR: estimated Glomerular Filtration Rate; FGF23: Fibroblast growth factor 23; PTH: parathyroid hormone; T50: Calcification propensity; Ln: log-transformed. (TIF) [file pone.0167929.s004.tif]

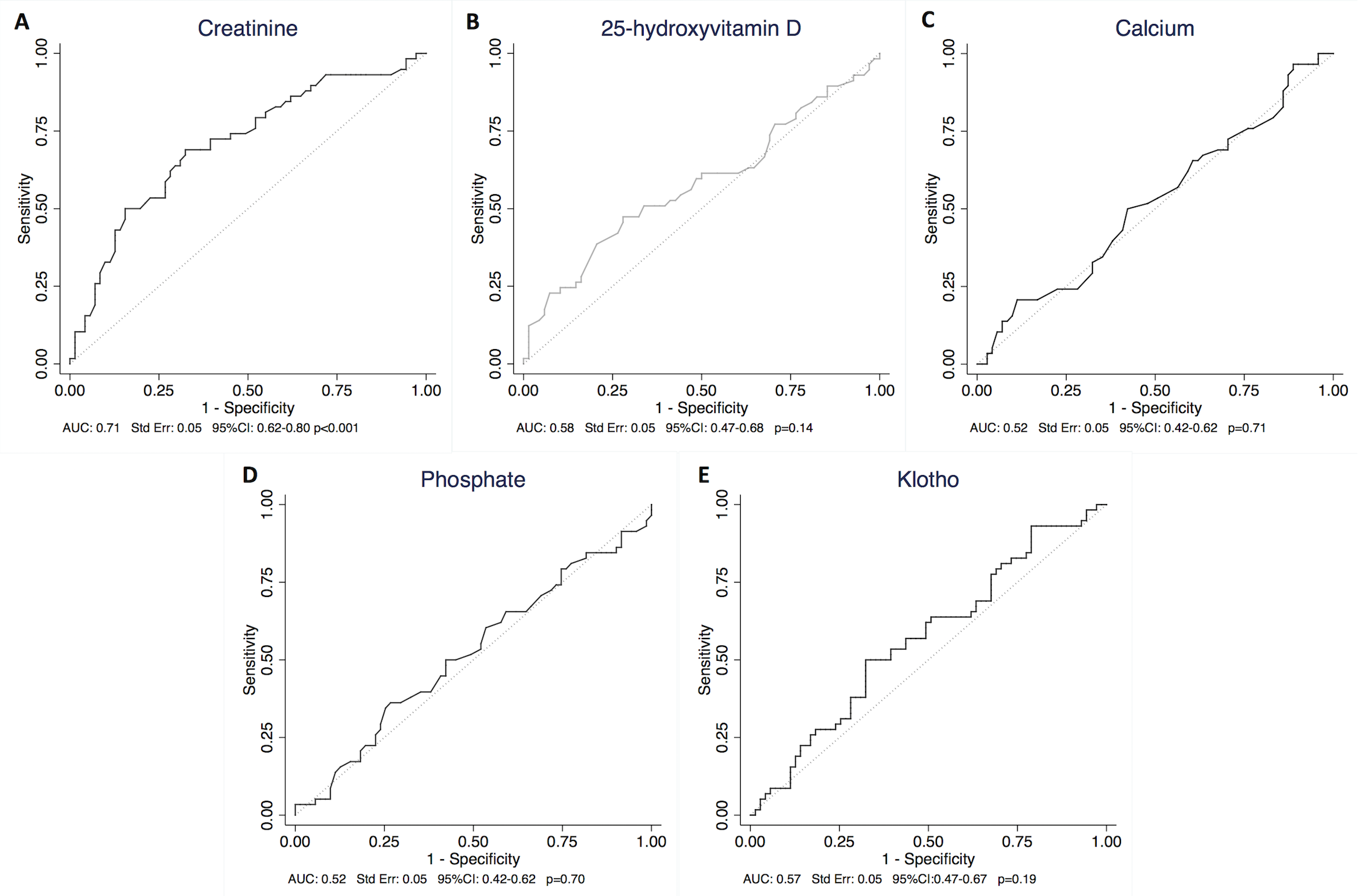

Supplement: S5 Fig — ROC curve analysis of creatinine (S5A Fig), vitamin D (S5B Fig), calcium (S5C Fig), phosphate (S5D Fig) and 5Klotho (S5E Fig) for the prediction of fibrosis ≤20%. As vitamin D and Klotho are markers that are negatively associated with fibrosis, we used the opposite values of those markers. AUC: Area Under the Curve; ROC: Receiver Operating Characteristic. (TIF) [file pone.0167929.s005.tif]

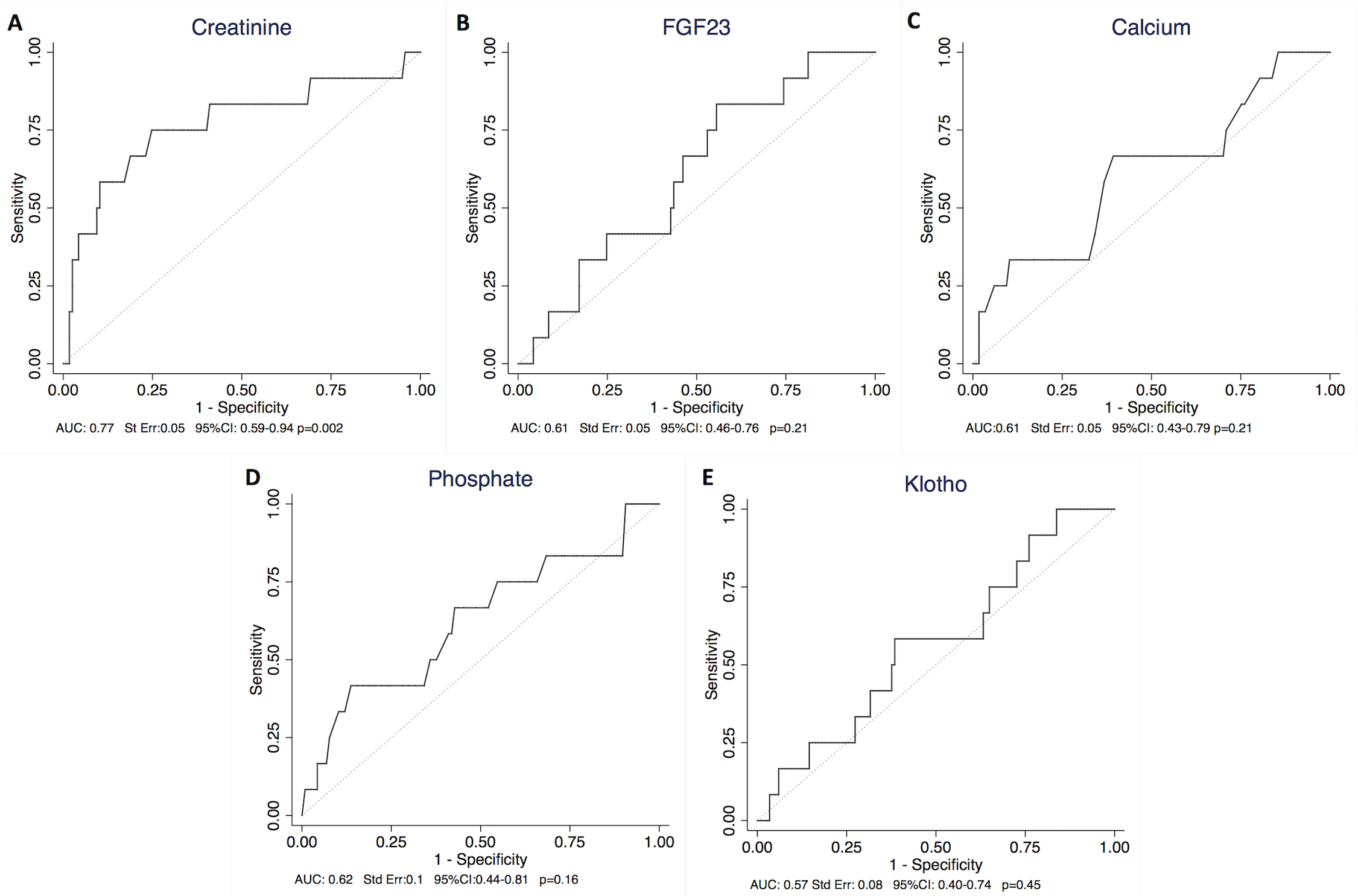

Supplement: S6 Fig — ROC curve analysis of creatinine (S6A Fig), vitamin D (S6B Fig), calcium (S6C Fig), phosphate (S6D Fig) and Klotho (S6E Fig) for the prediction of fibrosis >40%. As vitamin D and Klotho are markers that are negatively associated with fibrosis, we used the opposite values of those markers. AUC: Area Under the Curve; ROC: Receiver Operating Characteristic. (TIF) [file pone.0167929.s006.tif]

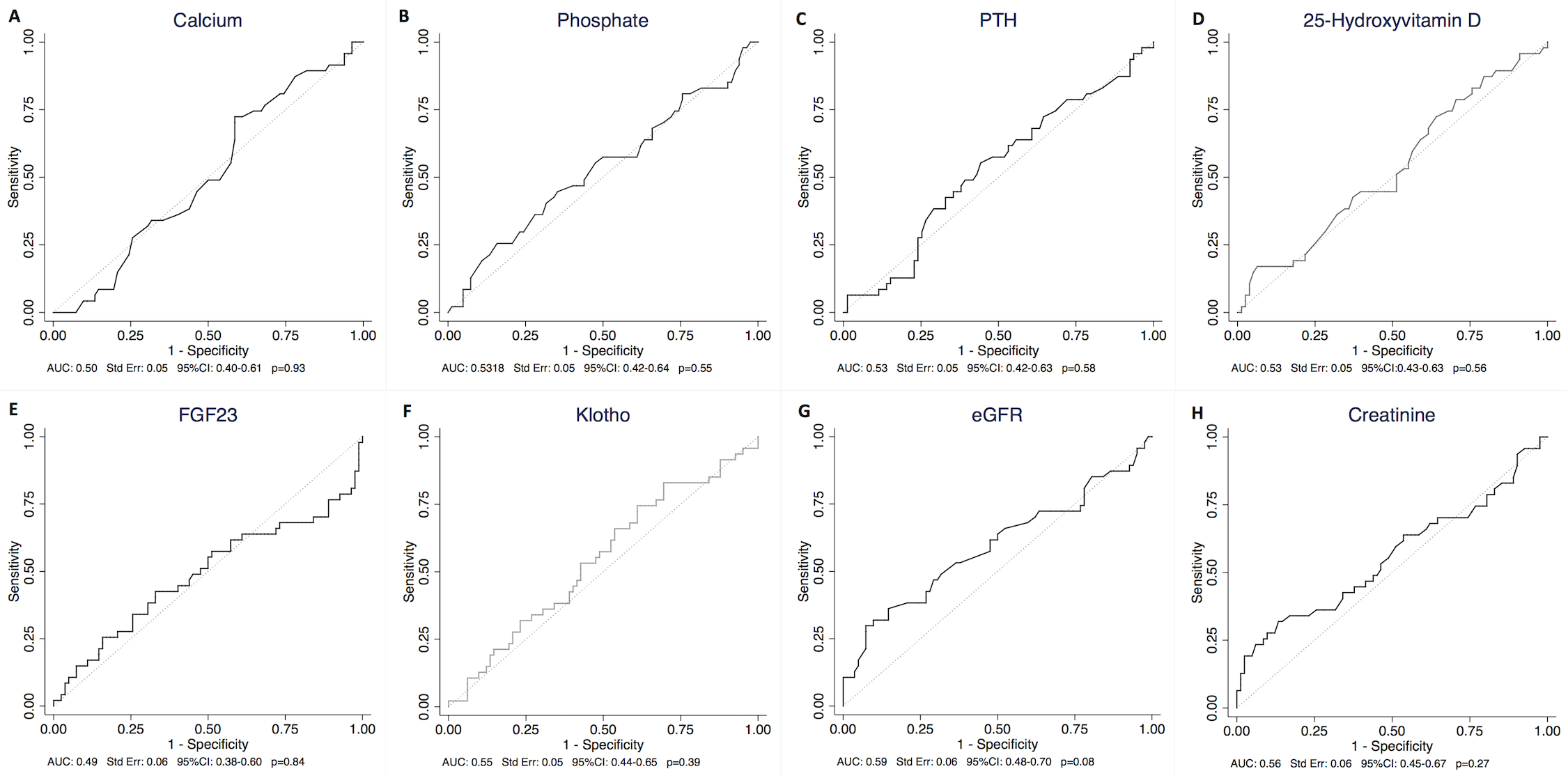

Supplement: S7 Fig — Vascular lesions estimated by BANFF: ROC curves of calcium (A), phosphate (B), PTH (C), vitamin D (D), FGF23 (E), Klotho (F), eGFR (G) and creatinine (H) in predicting significant vascular lesions (BANFF cv+ah+aah >5). Ah: arteriolar hyaline thickening; aah: circumferential hyaline arteriolar thickening; AUC: Area Under the Curve; cv: vascular fibrous intimal thickening; FGF23: Fibroblast growth factor 23; PTH: parathyroid hormone; ROC: Receiver Operating Characteristic. (TIF) [file pone.0167929.s007.tif]

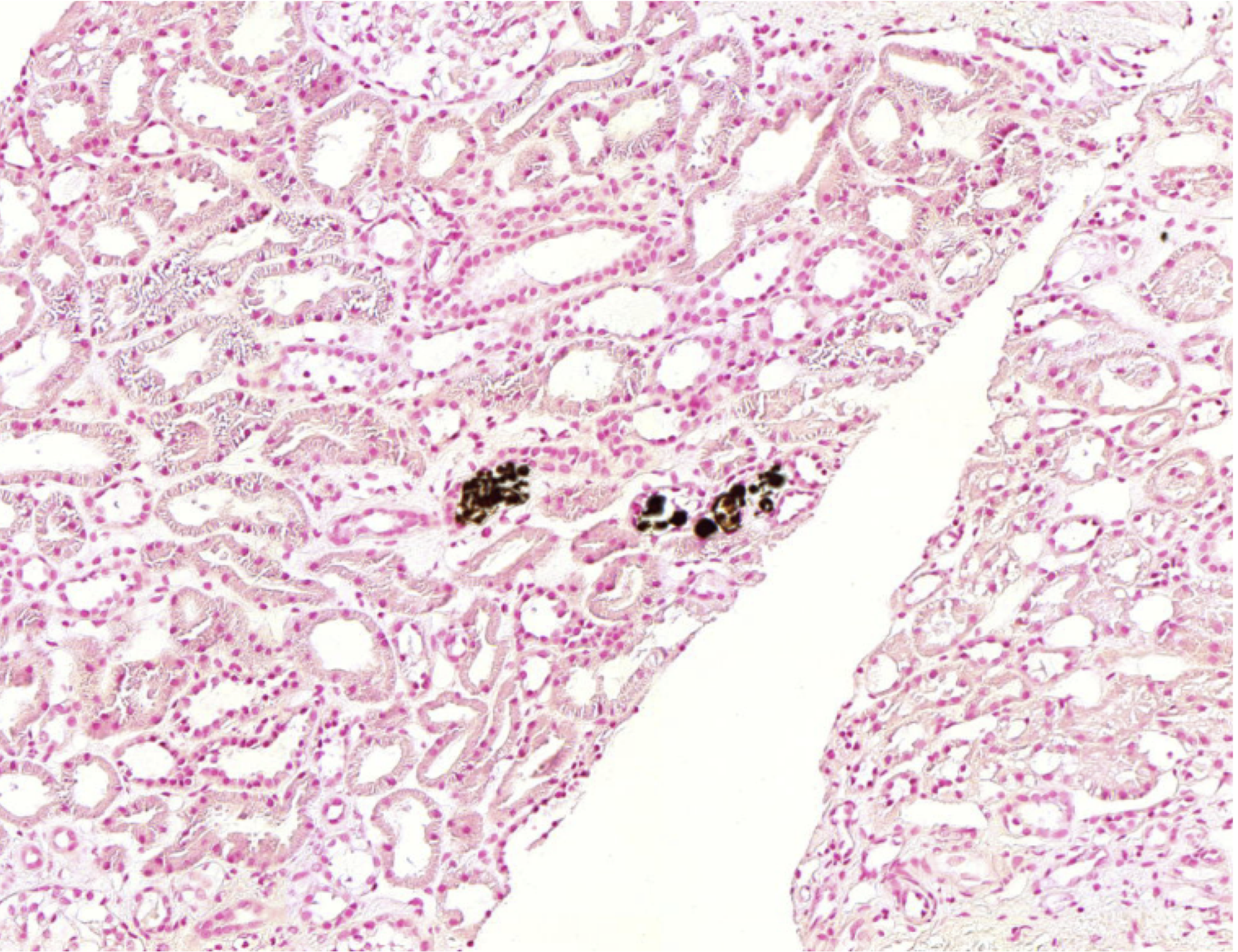

Supplement: S8 Fig — (TIF) [file pone.0167929.s008.tif]
