# Supplementary material for: Phosphocalcic Markers and Calcification Propensity for Assessment of Interstitial Fibrosis and Vascular Lesions in Kidney Allograft Recipients
Source: PLoS One. 2016 Dec 30;11(12):e0167929. doi: 10.1371/journal.pone.0167929 (PMC5201285; doi:10.1371/journal.pone.0167929)
Supplement: S1 Table — (PDF) [file pone.0167929.s009.pdf]

Supplementary Table

Table S1. Level values of phosphocalcic biomarkers and T_{50} across Von Kossa results (n=88).

Biomarkers	Positive Von Kossa (n=8)	Negative Von Kossa (n=80)	p Value
Phosphate (mmol/l)	0.96 (0.67-1.1)	1.11 (1.01-1.26)	0.14
Calcium (mmol/l)	2.39 (2.28-2.46)	2.43 (2.32-2.52)	0.17
Vitamine D (nmol/l)	62.5 (46.5-80)	70.5 (57-83)	0.31
PTH (pmol/l)	9.55 (6.35-16.6)	8.9 (6.2-10.8)	0.41
FGF23 (RU/ml)	29.5 (24.7-44.8)	39.3 (26.4-55.7)	0.26
Klotho (pg/ml)	731.7 (597.8-921.9)	695.0 (575.9-837.8)	0.73
T_{50} (min)	270 (232.5-318)	282 (247.5-330)	0.61
eGFR (ml/min/1.73m2)	50 (38-57.5)	53.5 (40.5-67)	0.38

Values reported as median and IQR. eGFR: estimated Glomerular Filtration Rate; FGF23: Fibroblast growth factor 23; PTH: parathyroid hormone; T_{50}: Calcification propensity.
